# Stem cell therapies in preclinical models of stroke associated with aging

**DOI:** 10.3389/fncel.2014.00347

**Published:** 2014-11-03

**Authors:** Aurel Popa-Wagner, Ana-Maria Buga, Thorsten R. Doeppner, Dirk M. Hermann

**Affiliations:** ^1^Department of Psychiatry, Aging and Brain Disorders, University of Medicine RostockRostock, Germany; ^2^Department of Functional Sciences, Center of Clinical and Experimental Medicine, University of Medicine and Pharmacy of CraiovaCraiova, Romania; ^3^Department of Neurology, University Hospital EssenEssen, Germany

**Keywords:** aging, stroke, cell therapy, comorbidities, neurogenesis

## Abstract

Stroke has limited treatment options, demanding a vigorous search for new therapeutic strategies. Initial enthusiasm to stimulate restorative processes in the ischemic brain by means of cell-based therapies has meanwhile converted into a more balanced view recognizing impediments related to unfavorable environments that are in part related to aging processes. Since stroke afflicts mostly the elderly, it is highly desirable and clinically important to test the efficacy of cell therapies in aged brain microenvironments. Although widely believed to be refractory to regeneration, recent studies using both neural precursor cells and bone marrow-derived mesenchymal stem cells for stroke therapy suggest that the aged rat brain is not refractory to cell-based therapy, and that it also supports plasticity and remodeling. Yet, important differences exist in the aged compared with young brain, i.e., the accelerated progression of ischemic injury to brain infarction, the reduced rate of endogenous neurogenesis and the delayed initiation of neurological recovery. Pitfalls in the development of cell-based therapies may also be related to age-associated comorbidities, e.g., diabetes or hyperlipidemia, which may result in maladaptive or compromised brain remodeling, respectively. These age-related aspects should be carefully considered in the clinical translation of restorative therapies.

## AGE AS MAJOR RISK FACTOR FOR STROKE

Stroke is a highly prevalent disease, which represents the second most common cause of death in Europe, and the third most common cause of death in the United States and Canada (Lloyd-Jones et al., 2010; Roger et al., 2012). Age is the principal non-modifiable risk factor for stroke. The incidence of stroke increases significantly with age in both men and women, with half of all strokes occurring in subjects over 75 years of age, and one-third in subjects over 85 years of age (Roger et al., 2012). Although the entire older population is at risk of stroke, there are important sex differences in stroke incidence. The incidence of stroke is higher in men than women up to 75 years old, equilibrates in subjects aged 75–84 years, and gets higher in women than men aged 85 years or older (Roger et al., 2012). This may be attributed to sex-related differences in risk factor profiles, resulting in a higher life expectancy in women than men. Importantly, age-associated changes show great variability among individuals, which are modified by genetic and long-term lifestyle factors (Tacutu et al., 2010, 2011).

## STROKE MODELS USING AGED ANIMALS ARE CLINICALLY MORE RELEVANT

Effects of age and sex on stroke incidence, functional neurological recovery and stroke mortality have been shown both in humans and in animal models (Badan et al., 2003; Buchhold et al., 2007; Gokcay et al., 2011). Specifically, the age-dependent acceleration in the progression of ischemic tissue into infarction strongly suggests that age is a biological marker for the variability in stroke outcome (Ay et al., 2005).

Over the past 10 years, a variety of models of middle cerebral artery occlusion (MCAO) have been established in rodents (Bacigaluppi et al., 2010). MCAO in aged rodents has been produced with permanent occlusion or transient occlusion for 30–120 min using (i) non-localized photothrombosis achieved by irradiation of the right carotid artery (Futrell et al., 1991); (ii) thermocoagulation after microcraniotomy (Davis et al., 1995); (iii) intraluminal using a silicon-coated suture (Sutherland et al., 1996); (iv) a hook attached to a micromanipulator (Popa-Wagner et al., 1998); (v) cauterization (Katsman et al., 2003); (vi) photothrombosis (Zhao et al., 2005); (vii) injection of a thrombus via the external carotid artery (Zhang et al., 2005, 2013; Dinapoli et al., 2006); or (viii) endothelin-1 injection (Soleman et al., 2010). These models differ in the localization and size of the ischemic lesions, as well as in mortality rates following surgery, as summarized in **Table 1**.

**Table 1:** Experimental stroke models and mortality rates in aged rats.

Strain	MCA occlusion	Age (mo)	Ischemia	Mortality (%)	Localization	Anesthetic	Reference
Wistar	Thermocoagulation	32	Permanent	NA	Neocortical	Halothane	Davis et al. (1995)
Wistar	Intraluminal nylon monofilament	27	Permanent or reversible	10	Necortical & striatal	Halothane	Sutherland et al. (1996)
Wistar	Photothrombosis	24	Permanent	4	Neocortical	Halothane	Zhao et al. (2005)
Fischer	Photothrombosis non-localized	24	Reversible	16	Neocortical	Chloral hydrate	Futrell et al. (1991)
Wistar	Photothrombosis	24	Permanent	4	Neocortical	Halothane	Zhao et al. (2005)
Wistar	Endothelin-1	20–23	Reversible	13.3	Neocortical	Isoflurane	Soleman et al. (2010)
Wistar	ECA embolization	18–20	Spontaneous reperfusion	33	Necortical & striatal	Isoflurane	Zhang et al. (2013)
Sprague-Dawley (m)	Vascular clip/hook	18–20	Reversible	25	Neocortical	Halothane	Popa-Wagner et al. (1998)
Sprague-Dawley (f)	ECA embolization	18	rt-PA reperfusion	33	Neocortical	Ketamine	Dinapoli et al. (2006)
Wistar	ECA embolization	18	Spontaneous reperfusion	47	Necortical & striatal	Halothane	Zhang et al. (2005)

*NA, not assessed; ECA, external carotid artery; f, female; m, male.*

Since focal cerebral ischemia is technically difficult to perform in very old rodents and since in humans stroke is highly prevalent in late middle aged (60–70 years old) subjects (Feigin et al., 2003), middle aged rodents may represent a reasonable choice for stroke studies (Popa-Wagner et al., 2007). In the following, specific responses of the aged brain to ischemic stroke and cell-based therapy are outlined. Results from cell transplantation studies in aged rodents are summarized in **Table 2**.

**Table 2:** Cell therapy studies in aged animals.

Cell type	Donor	Host	Age (mo)	Sex	Infarct	MCAO	Dose	Time after stroke	Route of administration	Reference
BM MSC	Rat, male	Wistar	11	Female	Neocortical & subcortical	Intraluminal suture occlusion	2 × 10^6^	24 h	Intra-carotid	Shen et al. (2007)
BM MSC	Rat	Long evans rat	12	Male	Neocortical	Cranioectomy, clipping	4–10 × 10^6^	24 h	Intra-carotid	Brenneman et al. (2010)
NPC	Human	Fischer 344 rat	24	Male	Neocortical	Cranioectomy, coagulation	1 × 10^5^	3 weeks	Infarct cavity	Jin et al. (2010a)
NPC	Human	Fischer 344 rat	24	Male	Neocortical	Cranioectomy, coagulation	1.2 × 10^5^	2 weeks	Infarct cavity	Jin et al. (2011)
BM MSC	Young SHR-SP rat	Old SHR-SP rat	15	Female	Neocortical	Cranioectomy, coagulation	0.5 × 10^5^	30 days before stroke	Intravenous	Taguchi et al. (2011)
UCTC	Human	Wistar rat	19	Male	Neocortical & subcortical	Embolization	1 × 10^7^	24 h	Intravenous	Zhang et al. (2013)
BM MSC	Rat	Sprague Dawley rat	19	Male	Neocortical	Cranioectomy, hook occlusion	1 × 10^6^	6 h	Intravenous	Balseanu et al. (2014)
iPSC	Human	Sprague Dawley rat	24	Male	Neocortical & subcortical	Intraluminal suture occlusion	0.3 × 10^6^	2 days	Intracortical	Tatarishvili et al. (2014)

*BM MSC, bone marrow-derived mesenchymal stem cells; NPC, neural precursor cells; UCTC, umbilical cord tissue cells; iPSC, inducible pluripotent stem cells.*

## SPONTANEOUS STROKE RECOVERY IN AGED PATIENTS AND ANIMALS

Neurological recovery is thought to occur via recruitment of neighboring neuronal circuitries (Hallett, 2001; Hermann and Chopp, 2012; Zhang et al., 2014). In clinical practice, physical therapy is used for stimulating post stroke brain remodeling (Liepert et al., 2004; Honmou et al., 2012).

Stroke patients regain some of their lost neurological functions during the first weeks or months after the stroke, most likely due to functional reorganization of the lesioned area (Zhang et al., 2014). In animal models of stroke, complete spontaneous recovery may occur in young–adult rats, depending on the size and location of the ischemic lesion. However, stroke recovery is delayed and often incomplete in aged rats. While young–adult rats typically begin to show improvements of neurological function starting by day 2 post stroke, neurological recovery is hardly detectable in aged rats before days 4 or 5, and achieve about 75% of the functional improvement observed in young–adult rats by day 14 (Buchhold et al., 2007). Housing experimental animals in an enriched environment enhances the recovery from brain damage both in young–adult and aged animals (Buchhold et al., 2007). When aged rats were allowed to recover in an enriched environment, the delay period was shortened and behavioral performance was significantly improved. The improvement in task performance positively correlated with slower infarct development, fewer proliferating astrocytes and smaller glial scars (Buchhold et al., 2007). Even more effective rehabilitation of the contralateral forelimb could be achieved by combining enriched environment with physical training (Hicks et al., 2007).

## STRATEGIES TO IMPROVE FUNCTIONAL NEUROLOGICAL RECOVERY AND TISSUE REPAIR AFTER STROKE BY CELL THERAPY

Although rehabilitation is important for improving functional recovery in the early stages after stroke, it does not provide a replacement of lost tissue. With this understanding, cell therapies have initially been implemented with the aim of replacing lost tissue in human stroke patients (Kondziolka et al., 2000; Bang et al., 2005).

Most clinical studies to date have used neural cells derived from human fetal donors. The techniques to achieve effective survival and growth of neural tissues transplanted into the CNS are meanwhile well established (Dunnett, 2013). Even though effective, neural grafting has not become a standard treatment for several reasons including the limited supply of fetal tissue of human origin and controversies about the beneficial effects (Morizane et al., 2008). Of the various options, the transplantation of adult stem and precursor cells, propagated in cell culture, or the use of inducible pluripotent stem cells (iPSCs) derived from human patients and trans-differentiated into neural cells, are reasonable alternatives (Haas et al., 2005; Stoll, 2014). iPSCs hold great promise for stroke treatment, since they lack ethical concerns and the risk of graft rejection. However, if and how the aged brain responds to grafted cells is still vaguely known unclear.

## NEURAL PRECURSOR AND STEM CELLS IN SUBCORTICAL STROKE

Spontaneous recovery is commonly observed if the infarct is located in the striatum, a subcortical structure that exhibits a natural activity-dependent plasticity. In animal models, neurological recovery is associated with structural dendritic and synaptic plasticity in the contralesional striatum (Qin et al., 2014) and with axonal plasticity in contralesional motor cortex (Reitmeir et al., 2012). This suggests that spontaneous recovery after a striatal stroke may also be augmented by inputs from contralateral striatum and could explain why patients with subcortical stroke are more likely to exhibit spontaneous functional neurological recovery (Rothrock et al., 1995; Bejot et al., 2008).

Cell-based therapy augments this endogenous response (Kokaia and Darsalia, 2011). Thus, human iPSCs implanted into striatum of young–adult animals at 1 week after MCAO, protected substantia nigra from atrophy, probably through a trophic effect via release of survival-promoting growth factors (Oki et al., 2012; Polentes et al., 2012; Tornero et al., 2013; Yuan et al., 2013).

How the cells are transplanted and where they are delivered are important issues in stem cell therapy. Data from many groups have shown that stroke increases the proliferation of endogenous neural precursor cells (NPCs) in the ipsilesional subventricular zone (SVZ) of young–adult rodents with a maximum at 1–2 weeks. These endogenous NPCs migrated along a scaffold of blood vessels to the peri-infarct striatum over a period of several months (Darsalia et al., 2007; Kojima et al., 2010). Some NPCs differentiated into medium size spiny neurons and may become part of the neuronal network (Arvidsson et al., 2002; Jin et al., 2006; Thored et al., 2006; Hou et al., 2008; Bacigaluppi et al., 2009; Doeppner et al., 2012; Mine et al., 2013). Noteworthy, the transplanted NPCs also stimulated neurogenesis in the ipsilateral SVZ and subgranular zone of the dentate gyrus (DG; Jin et al., 2011; Zhang et al., 2011; Mine et al., 2013).

In subcortical stroke, the location of the ischemic lesion in relation to the SVZ is thought to play a major role in stroke recovery. Unfortunately, the proportion of surviving neurons is discouragingly low (Arvidsson et al., 2002; Parent et al., 2002). Yet, the formation of neurons in the striatum is preserved in aged animals. Thus, the number of new striatal neurons in aged rodents after stroke was similar to that in young–adult rodents (Darsalia et al., 2005; Ahlenius et al., 2009), despite 50% decline in neurogenesis in the SVZ of elderly compared with young–adult animals (Enwere et al., 2004).

The subventricular cavity is lined up by ependymal cells that are quiescent and do not contribute to neurogenesis under normal conditions. However, in response to stroke ependymal cells enter the cell cycle and generate astrocytes and neuroblasts (Carlén et al., 2009). Signaling through the Notch pathway is required to maintain ependymal cell quiescence and suppresses neuronal differentiation and forced Notch signaling blocked the ependymal cell response to stroke (Carlén et al., 2009). However, in a more recent study ischemia-induced cell proliferation in the SVZ in aged rodents was enhanced by Notch1 activation and was associated with a reduced infarct volume and improved motor deficits (Wang et al., 2009; Sun et al., 2013). Conversely, ablation of doublecortin (DCX)-expressing cells with ganciclovir before MCAO in DCX-TK transgenic mice resulted in an increased infarct size and an adverse effect on functional outcome from cerebral ischemia (Jin et al., 2010b). Likewise, disruption of neurogenesis by low dose of irradiation, which, in part, inhibits DG neurogenesis, is associated with more severe functional impairments after cerebral global ischemia in gerbils (Raber et al., 2004).

Studies on post-mortem brains provided evidence for enhanced SVZ cell proliferation and neuroblast formation after stroke even in aged humans (Jin et al., 2006; Macas et al., 2006; Minger et al., 2007; Martí-Fàbregas et al., 2010). In line with the observation that new neurons are continuously formed in the adult human striatum (Ernst et al., 2014), an increased number of putative neuroblasts was noted in the human striatum in response to stroke (Macas et al., 2006).

However, whether endogenous neurogenesis contributes to spontaneous stroke recovery is still to be established. Human iPSC-derived long-term neuroepithelial-like stem cells (hiPSC-lt-NES) derived from a young adult had the potential to survive, differentiate into immature and mature neurons, and migrate to the peri-infarct tissue, when transplanted into the stroke-injured striatum and cortex in young–adult rats (Oki et al., 2012; Tornero et al., 2013). Young–adult rats treated with these cells showed improved neurological recovery as compared with animals not receiving such grafts (Oki et al., 2012; Tornero et al., 2013). Whether iPSC-lt-NES cells similarly promote neurological recovery in aged rats, when transplanted into subcortical brain areas, still remains to be shown.

## NEURAL PRECURSOR AND STEM CELLS IN CORTICAL STROKE

Cortical strokes lack the vicinity of a neurogenic niche like the SVZ, which impedes brain remodeling processes. Indeed, the post-acute delivery of adult mouse NPCs does not prevent secondary degeneration in the young–adult mouse cerebral cortex as much as the striatum, when animals are submitted to intraluminal MCAO (Bacigaluppi et al., 2009). Despite the less potent effects on neuronal survival, NPC delivery reduced glial scar formation in the surrounding of neocortical strokes (Bacigaluppi et al., 2009) and increased axonal plasticity in the contralesional motor cortex (Andres et al., 2011). These data convincingly show that the cerebral cortex does respond to restorative therapies.

If and how the aged brain responds to exogenous NPCs is poorly understood. The DG is one of the few brain regions to support neurogenesis in the adult by the recruitment of new granule cells into the hippocampal circuitry (van Praag et al., 2002). However, the extent of new granule cell recruitment is dramatically reduced in middle-aged (12 month-old) and aged (24 month-old) compared with young–adult (6 week-old) rats (Heine et al., 2004). The reduced precursor cell proliferation was not only caused by a general decline in total precursor cell numbers, but also by a reduced proliferation of the NPCs (Walter et al., 2011).

In animal models, focal neocortical infarcts induced proliferative changes that have been associated with an enhanced number of newborn neurons in the DG of young–adult but not aged mice (Walter et al., 2010). Therefore, later studies examined if exogenous NPC delivery might restore neurogenesis in the DG of old rodents. To test this hypothesis, NPCs isolated from embryonic caudal neural tubes of Sox-2:EGFP transgenic mice were expanded *in vitro* and bilaterally injected into the hippocampus of middle-aged (12 month-old) Fisher-344 rats (Hattiangady et al., 2007). Grafted NPCs migrated to all layers of the hippocampus and enhanced the number of new dentate granule cells. Increased dentate neurogenesis, measured by DCX staining in the aging hippocampus following grafting likely reflected a more conducive microenvironment for proliferation of endogenous NPCs in the presence of grafts of exogenous NPCs (Hattiangady et al., 2007). In another study, intracerebroventricular administration of conditionally immortalized human fetal brain (CTX0E03) cells in 22 month-old rats, stimulated the proliferation of NPCs in the subgranular zone of the DG as demonstrated by immunohistochemical staining for the immature neuronal marker DCX (Park et al., 2010).

Most previous studies of NPC transplantation in rodents have employed post-ischemic intervals of 1 week or less. However, the option for delayed treatment is clinically important. In aged (24 month-old) Sprague-Dawley rats, transplantation of human embryonic stem cell (hESC)-derived NPCs integrated into Matrigel scaffolds reduced ischemic infarct volume and improved neurological recovery even when implanted into the cavity of neocortical infarcts as late as 3 weeks after stroke (Jin et al., 2010a). In a subsequent study, the transplantation of human NPCs together with Matrigel into the lesioned neocortex of young adult (3 month-old) and aged (24 month-old) male Fisher-344 rats at 2 weeks after stroke stimulated neurogenesis in the SVZ ipsilateral to stroke, as demonstrated by increased numbers of cells expressing the early neuronal lineage marker Dcx at 60 days post-transplantation (Jin et al., 2011).

In a recent study, human iPSC transplanted directly into the damaged neocortex of aged rats survived, differentiated into neurons and improved functional recovery in cylinder test at 4 and 7 weeks (Tatarishvili et al., 2014). The grafted hiPSC suppressed microglia/macrophage activation in the stroke-injured cortex as evidenced by differential morphological changes of these cells in the cell-grafted and vehicle-injected animals (Tatarishvili et al., 2014). Although it is not clear how microglia/macrophages were affected at earlier time-points after stroke, it seems possible that the observed immunomodulatory action of the grafts could contribute to both neuroprotective and plasticity responses. Consistent with our findings, previous studies have shown that human fetal NPCs transplanted into cortex or striatum reduced the number of microglia/macrophages in the peri-infarct tissue 1, 6, and 14 weeks after stroke (Horie et al., 2011; Mine et al., 2013). Similarly, inhibition of microglial activation has also been reported in ischemic mice following systemic delivery of mouse NPCs (Bacigaluppi et al., 2009).

Inducible pluripotent cells generated from human fibroblasts of aged humans may be differentiated into specific cell types, namely into functional motor cortical neurons (Mohamad et al., 2013; Phanthong et al., 2013). Interestingly, the differentiation capacity into motor cortical neurons was the same for iPSCs obtained from 29 and 82 year-old individuals (Boulting et al., 2011).

## MESENCHYMAL STEM CELLS IN STRIATAL AND NEOCORTICAL STROKE

Patients with cerebrovascular diseases have decreased numbers of circulating bone marrow-derived CD34+ precursor cells, which have been suggested to have prognostic value for neurologic function in patients with history of brain infarction (Taguchi et al., 2009). These findings have prompted experiments aiming at the restoration of circulating bone marrow-derived precursor cells in stroke models by transplantation of autologous hematopoietic progenitor cells.

Mesenchymal stem cells (MSC) derived either from bone marrow or adipose tissue have repeatedly been shown to ameliorate neurological recovery in experimental stroke models (Schwarting et al., 2008; Honmou et al., 2012; Kocsis and Honmou, 2012). When administered in the acute stroke phase, MSCs decreased infarct volume, improved neurological function, enhanced neurogenesis, and exerted anti-inflammatory actions. MSCs enhance recovery processes in the injured brain by promoting angiogenesis, neurogenesis, and neural reorganization (Hayase et al., 2009; Bao et al., 2011; Lim et al., 2011; Hsieh et al., 2013).

Improved neurological recovery associated with preservation of pyramidal tract axons ipsilateral to the stroke have also been described in 12-month-old ischemic rats systemically treated with bone marrow-derived MSCs (Shen et al., 2007). Neurological improvements persisted for at least 1 year (Shen et al., 2007). When delivered to 12-month-old ischemic rats, the improvement of neurological recovery and reduction of infarct volume induced by autologous bone marrow-derived MSC transplantation resembled that in 2–3-month-old rats (Brenneman et al., 2010), indicating that aging does not impair the responsiveness to MSC therapy. This was particularly noteworthy as the number of transplanted cells in the peri-infarct tissue decreased within hours and were almost undetectable after 7 days (Brenneman et al., 2010).

Similar to bone marrow-derived cells, umbilical cord-derived blood cells (UCBCs) are widely available, representing an attractive source for cell-based therapies. Human UCBCs are rich in mesenchymal and endothelial precursor cells and can be collected without the ethical concerns associated with embryonic or fetal cells. Intravenous injection of human UCBCs in aged (20-month-old) rats restored the age-related decrease of endogenous neurogenesis and reduced brain inflammation (Bachstetter et al., 2008). In young–adult rats exposed to focal cerebral ischemia, intravenous delivery of CD34+ UCBCs enhanced endogenous angiogenesis, neurogenesis and functional neurological recovery in some (Chen et al., 2001; Taguchi et al., 2004a,b), but not other (Mäkinen et al., 2006) studies. In aged (18–20-month-old) rats exposed to focal cerebral ischemia, intravenous delivery of human umbilical tissue-derived cells improved functional neurological recovery and increased angiogenesis and synaptogenesis (Zhang et al., 2013). This suggests that umbilical cells may be used for stroke treatment in the aged ischemic brain.

Based on these observations, further clinical studies using intravenously delivered MSCs have been initiated in human stroke subjects (Lee et al., 2010; Moniche et al., 2012). Transplantation of autologous bone marrow-derived MSCs in pilot studies has been proven safe in human patients with acute middle cerebral artery infarcts (Bang et al., 2005; Battistella et al., 2011; Savitz et al., 2011; Friedrich et al., 2012; Moniche et al., 2012; Banerjee et al., 2014). Moreover, improvements of neurological recovery were noted (Bang et al., 2005; Battistella et al., 2011; Savitz et al., 2011; Friedrich et al., 2012; Moniche et al., 2012; Banerjee et al., 2014). Unfortunately, these studies lacked appropriate control groups, which limits conclusions around the efficacy of cell therapy.

In the translation of studies from bench to bedside, care should be taken that not only aging, but also age-related co-morbidities may affect brain remodeling and responses to cell-based therapies. Thus, the delivery of bone marrow-derived MSCs did not improve neurological recovery in rats exhibiting streptozotocin-induced type I diabetes, but increased mortality, blood–brain barrier leakage and brain hemorrhage (Chen et al., 2011). Besides, excessive angiogenesis was noted in diabetic rats receiving MSCs that was associated with cerebral arteriole narrowing and neointima formation inside the internal carotid artery (Chen et al., 2011). In histochemical studies, increased macrophage accumulation was noted in blood vessels of diabetic MSC treated rats. The authors suggested that MSC treatment should not be considered in diabetic patients.

Not only type I diabetes, but also hyperlipidemia may affect post-ischemic brain remodeling, as shown in studies showing the attenuation of VEGF-induced angiogenesis in ischemic ApoE-/- mice receiving Western diet (Zechariah et al., 2013), presumably via internalization and degradation of VEGF receptor-2 (Jin et al., 2013). Further studies identifying the conditions of efficacy and safety of cell-based therapy under conditions of vascular risk factors and diseases are warranted.

## CO-TRANSPLANTATION STRATEGIES AND COMBINATION THERAPIES

Poor survival and differentiation of both the transplanted cells and their progenies in the inhospitable environment of the infarcted cortex has prompted the search for alternatives and new concepts like the neurovascular unit to limit the loss of transplanted cells. To this end cotransplantation of endothelial cells with NPCs in a mouse model of stroke enhanced the survival, proliferation, and differentiation of transplanted cells and improved functional neurological recovery (Nakagomi et al., 2009). Even more challenging, cotransplantation of mouse embryonic stem cell-derived vascular progenitor cells (VPCs) with NPCs after ischemic stroke produced not only neural cells but also endothelial cells and pericytes, thus providing nearly all important components for recovery of the neurovascular unit, i.e., neurons, astroglia, endothelial cells, and mural vascular cells (Li et al., 2014).

Granulocyte colony stimulating factor (G-CSF) has been particularly successful when used to improve neurological function in various types of focal cerebral ischemia in young–adult animals (Shyu et al., 2004; Schäbitz and Schneider, 2007). We have recently been able to show that G-CSF treatment in 20-month-old aged rats enhances animal survival, improved functional neurological recovery, and induced neurogenesis in the ipsilesional SVZ (Popa-Wagner et al., 2010). Next, we reasoned that the efficiency of the bone marrow-derived-cell therapy may be increased by simultaneous application of G-CSF. In particular, we tested the hypothesis that grafting of pre-differentiated bone marrow-derived MSCs in G-CSF-treated animals would improve long term functional outcome in aged rodents (Balseanu et al., 2014). Although the combination therapy significantly improved neurological recovery and increased microvessel density in the former infarct core, neither G-CSF nor the combination decreased animal mortality, reduced infarct volume or increased neurogenesis (Balseanu et al., 2014).

**FIGURE 1:**
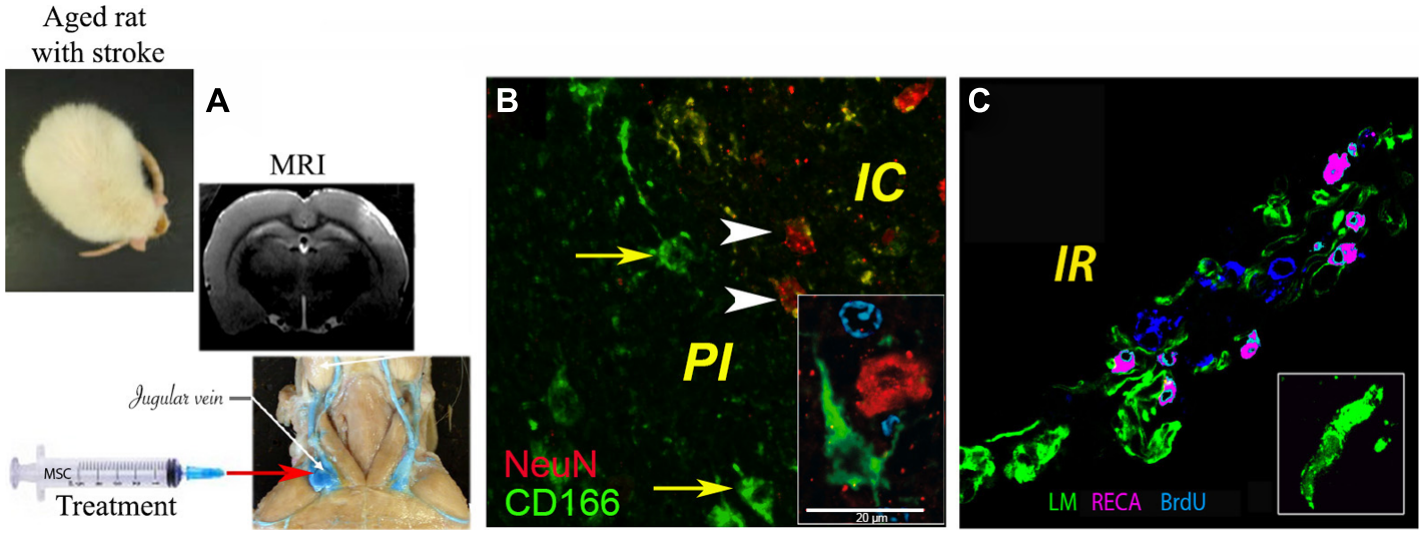
**Delivery of bone marrow-derived MSCs in aged rats exposed to focal cerebral ischemia. (A)** Administration of MSCs in the acute stroke phase via the jugular vein, brain infarcts being detected by MRI; **(B)** histochemical analysis of CD166+ cells (green) in the infarcted area, where ∼1% of injected MSCs were found intermingled with NeuN+ neurons (green); **(C)** angiogenesis as revealed by rat endothelial cell antigen (RECA; purple)/BrdU (blue) immunostaining. Basal laminae were counterstained with antibody against laminin (LM; green; modified based on Balseanu et al., 2014). Scale bar, 20 μm.

The mechanisms by which MSCs may ameliorate infarcted brain tissue seem related more to the capacity of MSCs to release neuroprotective factors (paracrine action) than to their capacity to replace damaged neural cells. Thus, around the former infarct core, several groups noted vigorous angiogenesis as evidenced by BrdU-positive endothelial cells (reviewed in Bronckaers et al., 2014). In our study, the density of the newly formed blood vessels was significantly higher in the brains of aged animals treated with the combination of G-CSF and MSCs as compared to controls and G-CSF, but not MSCs alone (Balseanu et al., 2014; **Figure 1**).

## CONCLUSION

Taken together,

(i) the restorative potential of the brain is preserved in aged, ischemic animals, although specific age-related aspects appear to exist related to the accelerated progression of infarction, the decreased proliferation of endogenous NPCs and the delayed initiation of neurological recovery (Zhang et al., 2013; Balseanu et al., 2014),
(ii) the environment of the aged brain does not preclude effects of grafted NPCs, MSCs, or iPSCs on brain remodeling, endogenous neurogenesis and functional neurological recovery,
(iii) detrimental consequences may result from age-related vascular co-morbidities, namely from diabetes or hyperlipidemia, in which maladaptive or impaired brain remodeling has been described,
(iv) although endogenous neurogenesis has been observed, even in aged humans, questions remain regarding the functional relevance of newly formed neurons for stroke recovery in human patients, where distances between endogenous stem cell niches and stroke injuries are so much larger due to the bigger size of the brain. Further research must explore optimal transplantation protocols (Dailey et al., 2013). Fascinating possibilities have recently been identified in the transdifferentiation capacity of iPSCs to specific neuronal subtypes, which might open new perspectives for cell replacement therapy. Whether such strategies may at all contribute to functional neurological recovery, remains unclear.

## Conflict of Interest Statement

The authors declare that the research was conducted in the absence of any commercial or financial relationships that could be construed as a potential conflict of interest.
